# Technique for Lateral Opening Wedge Distal Femoral Osteotomy Using Patient‐Specific Instrumentation for Genu Valgum and Patellar Instability

**DOI:** 10.1002/atn2.70081

**Published:** 2026-04-30

**Authors:** Alexander P. Decilveo, Elan Karlin, Neil Patel, Allison Ariniello, George F. Rick Hatch

**Affiliations:** ^1^ Department of Orthopaedic Surgery Southern California Permanente Medical Group Los Angeles California U.S.A.; ^2^ Department of Orthopaedic Surgery Keck School of Medicine of USC Los Angeles California U.S.A.; ^3^ Department of Orthopaedic Surgery St. Joseph's Regional Medical Center Paterson New Jersey U.S.A.

## Abstract

Recurrent lateral patellar instability poses a technical challenge to orthopaedic surgeons. Isolated soft‐tissue procedures through medial patellofemoral ligament reconstruction or lateral release show unacceptably high failure rates. Lateral opening wedge distal femoral osteotomy addresses underlying bony malalignment and reinforces the soft‐tissue procedure. Patient‐specific instrumentation has shown utility in the setting of osteotomies with improved accuracy and reduction of intraoperative hinge fractures.

## Graphical abstract

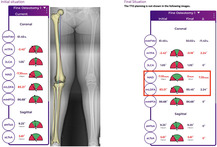

**VIDEO 1:** Technique for performing lateral opening wedge distal femoral osteotomy using patient‐specific instrumentation generated with BodyCad (Quebec City, CA) software. Right knee, supine positioning, with exam under anesthesia and diagnostic arthroscopy for patellar instability with use of the superolateral portal. The right distal femur is exposed, patient‐specific cutting guide is appropriately positioned, and osteotomy cut is performed with fluoroscopic guidance. Anterior and posterior wedge validators are positioned at the osteotomy site to allow for patient‐specific plate placement. The plate is secured with a patient‐specific drill guide for appropriate screw placement. Video content can be viewed at https://doi.org/10.1002/atn2.70081.

Lateral patellar dislocations have a cited incidence of 23.3 per 10,000 persons annually.^1^ First‐time dislocations are routinely treated nonoperatively, but recurrence rates after nonoperative management have been cited as high as 15% to 44%.^2^ Medial patellofemoral ligament reconstruction (MPFL‐R) significantly reduces redislocation rates, yet dislocation events persist with a rate of 2% to 11%.^3^ Patients who suffer from patellar instability often have underlying anatomic abnormalities that contribute to their pathology. These risk factors may include trochlear dysplasia, patella alta, enlarged tibial tubercle‐trochlear groove distance, and coronal and/or rotational malalignment. Workup for recurrent patellar instability has expanded to evaluation of weight‐bearing, full length radiographs, and advanced imaging torsional analysis with treatment strategies involving osteotomy to normalize anatomic bony deformities.

In patients who fail isolated MPFL‐R, underlying bony abnormalities must be evaluated. In patients with genu valgum and lateral patellar instability, lateral opening wedge distal femoral osteotomy (LOW‐DFO) has been shown to be an appropriate intervention, neutralizing the inappropriate force vector on the patella and restoring patellofemoral contact forces.^1^, ^4^, ^5^

Although freehand osteotomy techniques provide a standardized methodology, orthopaedic surgeons have found utility in the use of patient‐specific preoperative planning software, custom‐made 3‐dimensional (3D) cutting guides, and plates that are tailored to patients’ anatomy. Patient‐specific instrumentation (PSI) has shown improved accuracy of osteotomy cuts, precise gap correction, and reduced rates of intraoperative hinge fractures.^6^, ^7^, ^8^, ^9^ This article describes our technique for LOW‐DFO using PSI with concomitant MPFL‐R, and tibial tubercle osteotomy (TTO).

## SURGICAL TECHNIQUE

### Preoperative Deformity Assessment

**FIGURE 1:**
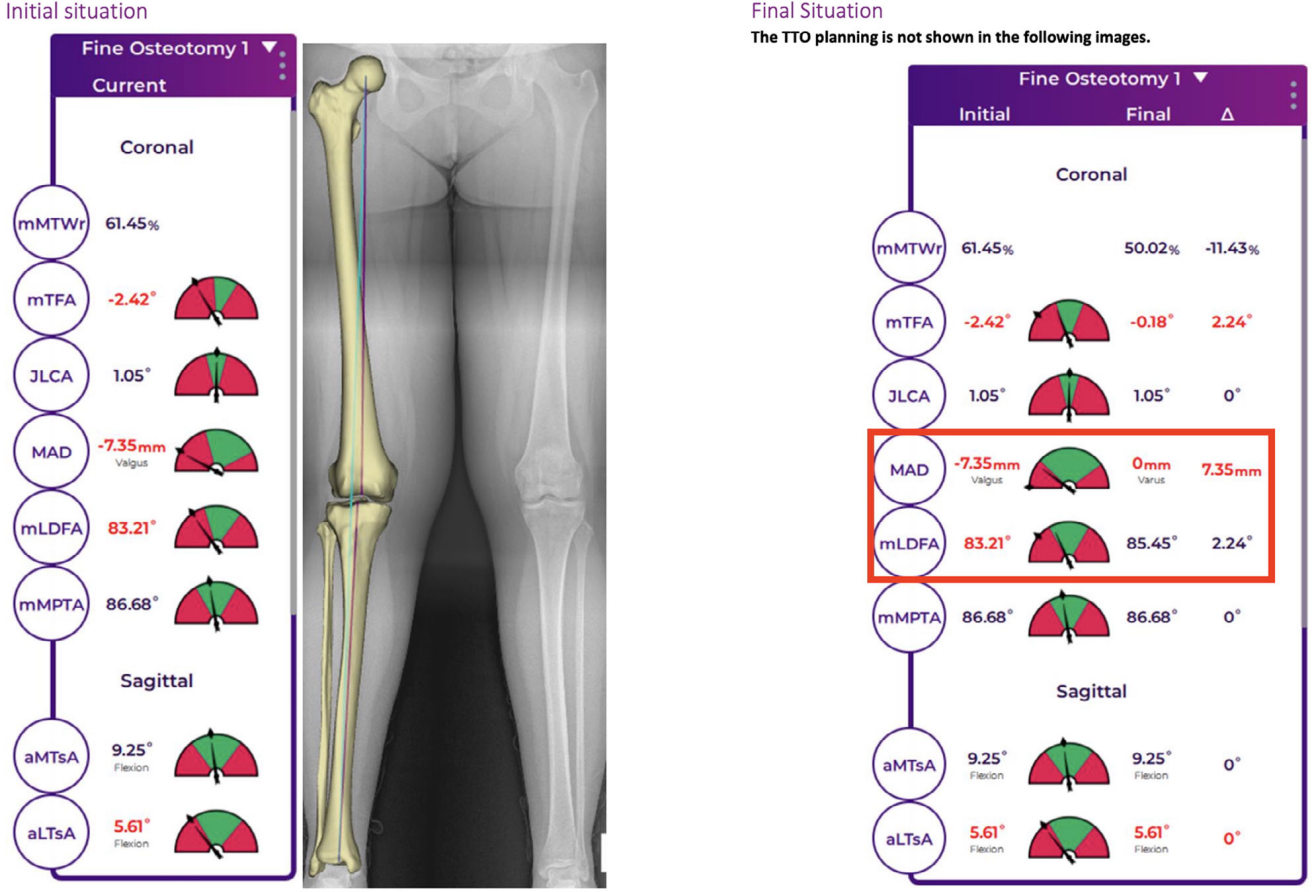
Patient‐specific preoperative plan. BodyCad (Quebec City, CA) patient‐specific software, based on preoperative bilateral lower extremity CT scanograms, analyzes the coronal and sagittal parameters for right lower extremity mechanical alignment of a patient with a valgus deformity of the right knee. The patient has an initial lateral distal femoral angle (mLDFA) measuring 83.21° and lateral MAD of 7.35 mm (red box). The software displays the degree of correction and final alignment parameters with a 3.1 mm opening gap osteotomy, based on the preoperative generated plan that normalizes the patient's mLDFA to 85.45° and MAD to neutral (red box). (aLTsa, anatomic lateral tibial slope angle; aMTsA, anatomic medial tibial slope angle; CT, computed tomography; JLCA, joint line congruence angle; MAD, mechanical axis deviationm; mLDFA, mechanical lateral distal femoral angle; mMPTA, mechanical medial proximal tibial angle, mTFA, mechanical tibiofemoral angle; MTWr, mechanical medial tibial width ratio).

BodyCad (Quebec City, CA) patient‐specific software is used to perform a preoperative assessment of mechanical alignment deformity based on preoperative bilateral lower extremity computed tomography scanograms (Figure 1). The software measures the degree of valgus deformity based on lateral distal femoral angle measurements, mechanical axis deviation (MAD), and various other parameters. A corrective opening gap osteotomy cut is generated that will address the patient's deformity and restore their MAD to neutral. An MPFL‐R and TTO may be indicated depending on the patient's anatomy and should be performed during the same surgery as the DFO. The sequence for the surgery recommended by the authors is as follows: First, the MPFL femoral origin is located under fluoroscopy, and the femoral tunnel is drilled. Next, a diagnostic knee arthroscopy is performed using a 70° arthroscope, followed by a lateral opening wedge DFO. Lastly, the TTO is performed followed by MPFL‐R.

### Patient Positioning and Exam Under Anesthesia

**FIGURE 2:**
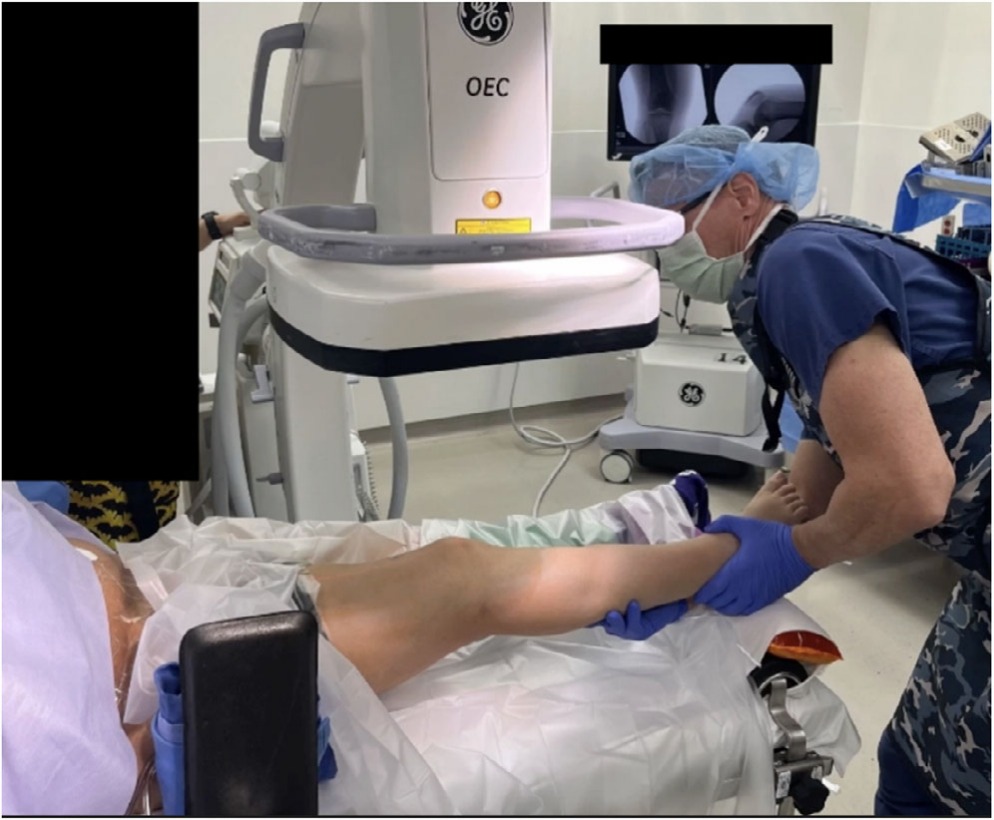
Patient positioning. Right knee, proximal to the left, distal to the right. The patient is placed supine on the operative table with a high thigh tourniquet, lateral hip post, and foot bump. If preferred, a pediatric bone foam can be placed in place of the foot bump to allow for resting 35° of knee flexion. A full‐size fluoroscopic unit enters from the contralateral or left side of the patient to obtain preoperative AP and lateral radiographs. (AP, anteroposterior.)

The patient is placed supine on the operative table with a high thigh tourniquet, lateral hip post, and foot bump (Video 1). If preferred, a pediatric bone foam can be placed in place of the foot bump to allow for resting 35° of knee flexion. A full‐size fluoroscopic unit enters from the contralateral side and preoperative anteroposterior (AP) and lateral fluoroscopic images are obtained (Figure 2). Bedside Doppler assessment is performed of the posterior tibial and dorsalis pedis arteries. Preoperative femoral and sciatic nerve catheters are placed. Exam under anesthesia is performed to assess for lateral patellar instability.

### Diagnostic Arthroscopy for Patellar Instability

Intravenous tranexamic acid is given, and the tourniquet is inflated. After creation of standard anterolateral and anteromedial arthroscopic portals, a spinal needle is used to create an accessory superolateral portal in the suprapatellar pouch. A 70° scope is placed in the superolateral portal, and a dynamic arthroscopic exam under anesthesia is performed in various knee flexion angles to assess patellar instability. In this patient, the patella could be fully dislocated until 45° of knee flexion. The arthroscope is removed and knee suctioned dry.

### Lateral Distal Femoral Exposure and Dissection of Kaplan Fibers

Next, a lateral distal femoral incision is made centered over the iliotibial band (IT) band down to the lateral epicondyle of the femur. Skin flaps are elevated anteriorly and posteriorly. The IT band is split in line with its fibers using a 15 blade and extended proximally and distally with Metzenbaum scissors. Electrocautery is used to elevate the vastus lateralis off the distal femur. Use caution when cauterizing arterial contributions from the superior lateral geniculate artery posteriorly and do not violate the intermuscular septum.

**FIGURE 3:**
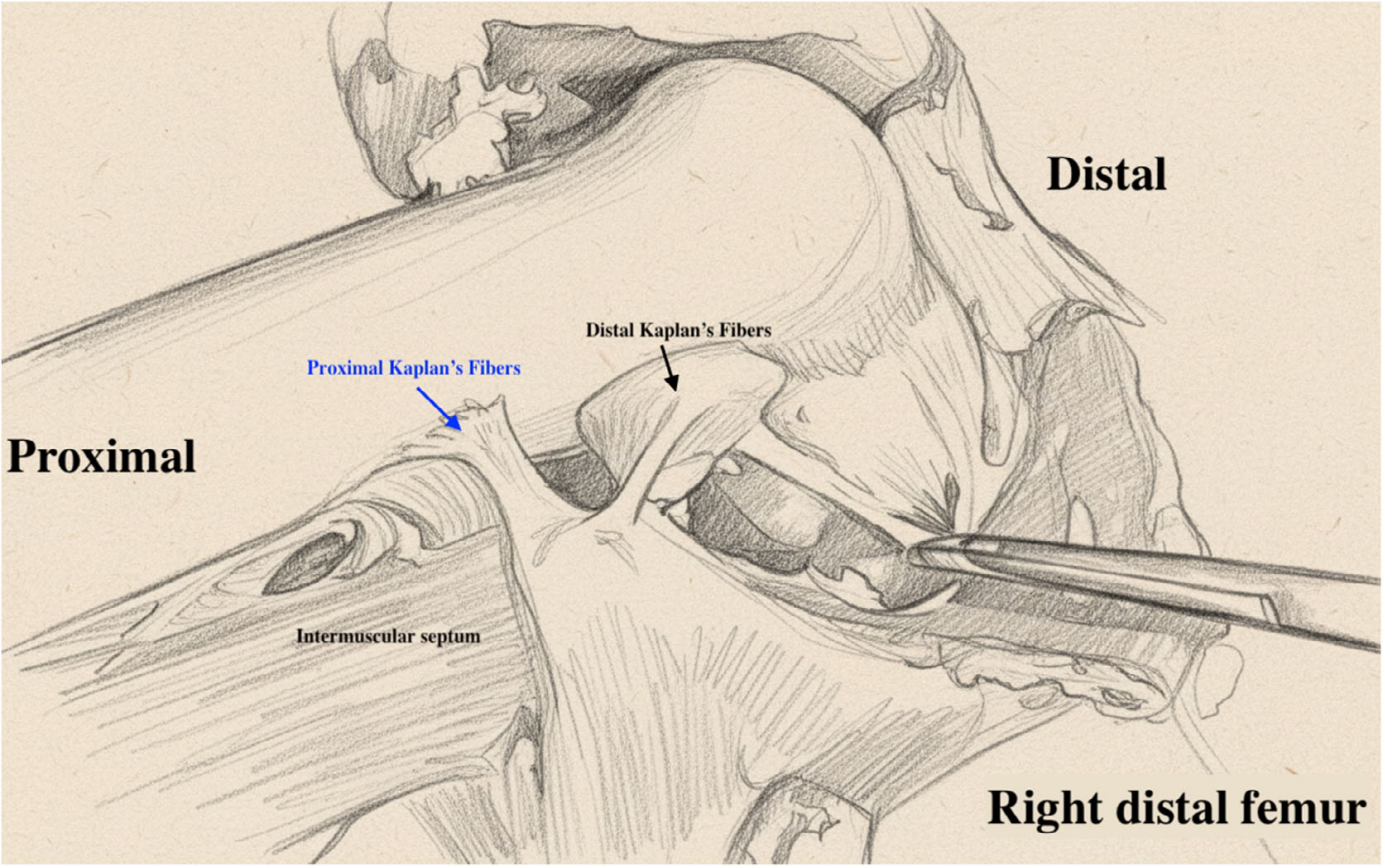
Animation of proximal and distal Kaplan fibers. Right lower extremity distal femur, proximal to the left, distal to the right. Both proximal (blue arrow) and distal (black arrow) Kaplan fibers can be seen along the posterolateral border of the distal femur.

Radiolucent Hohmann retractors are placed on the anterior and posterior aspects of the distal femur. An Army‐Navy retractor is used proximally to apply traction to the vastus lateralis muscle fibers and distally to retract the IT band. Dissection is carried distally until Kaplan fibers are elevated off the distal femur (Figure 3). This step is crucial as the patient‐specific cutting guide will not fit properly if proximal and distal Kaplan fibers are not elevated off the distal femur.

### Patient‐Specific Cutting Guide Placement

**FIGURE 4:**
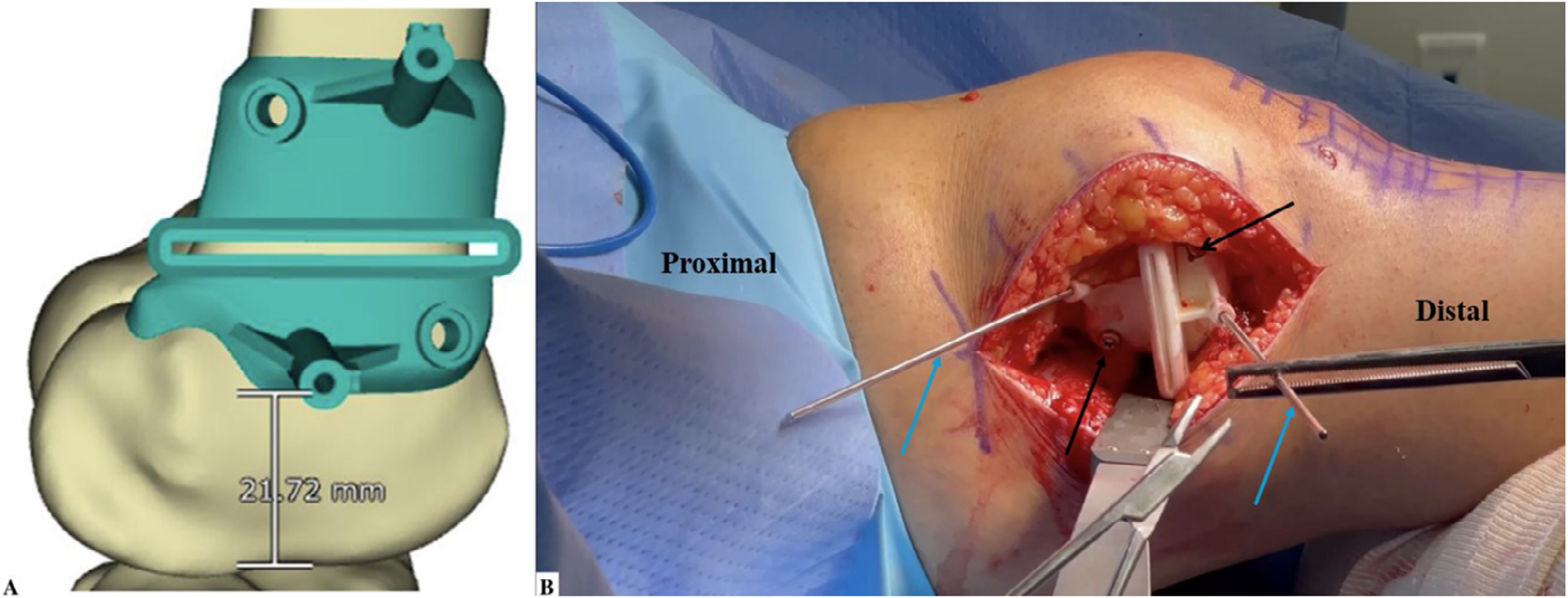
(A,B) Placement of 3D patient‐specific cutting guide. Right knee, supine position, proximal to the left, distal to the right. According to the BodyCad (Quebec City, CA) preoperative plan, the 3D patient‐specific cutting guide is placed along the lateral distal femur approximately 21.7 mm from the joint line (A) and secured with temporary holding set screws (black arrows) and 2 2.4 mm Kirschner wires (blue arrows) (B). (3D, 3‐dimensional.)

According to the BodyCad (Quebec City, CA) software‐generated 3D sterile patient bone model, intraoperative 3D plan, and bony landmarks, a patient‐specific cutting guide is developed and placed along the lateral distal femur approximately 21 mm from the joint line (Figure 4). Two 2.4 mm hinge pins are drilled nearly bicortically and 2 set screws are placed in the guide. Position of the guide and hinge pins are confirmed on AP and lateral fluoroscopic views and compared with the 3D computer‐generated model. The hinge pins are then cut shorter to facilitate saw entry.

### Osteotomy With Oscillating Saw

**FIGURE 5:**
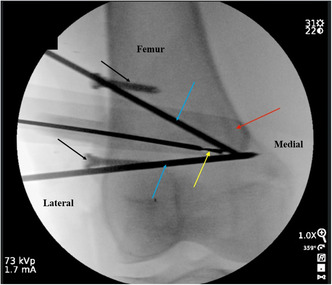
Placement of radiolucent retractor for osteotomy. Right knee, anteroposterior fluoroscopic image taken with patient in supine position. Temporary holding set screws (black arrows) and 2.4 mm Kirschner wires (blue arrows) secure the 3D patient‐specific cutting guide on the lateral aspect of the knee as the oscillating saw (yellow arrow) is introduced through the cutting guide. Position of the wide radiolucent retractor placed posteriorly behind the distal femur (red arrow) should be in line with the direction and position of the oscillating saw (yellow arrow) on AP fluoroscopic radiographs as it is crucial for protecting the posterior neurovascular structures. (3D, 3‐dimensional; AP, anteroposterior.)

The saw blade is marked to the proper depth for the osteotomy cut according to the 3D plan. A wide radiolucent Hohmann retractor is placed posteriorly to protect the neurovascular structures. An additional radiolucent Hohmann retractor is placed anteriorly to protect the muscle. Position of the posterior retractor in line (or parallel) with the osteotomy cut is crucial and confirmed on AP fluoroscopic views prior to cutting (Figure 5). The osteotomy is completed according to the depth marked on the saw and confirmed with fluoroscopy. Irrigation is used intermittently to reduce the heat of the saw blade. The saw blade is then removed.

### Remove Patient‐Specific Cutting Guide and Finish With Osteotome

The patient‐specific cutting guide and hinge pins are removed. Anterior and posterior Hohmann retractors are placed again, and the osteotomy is gently completed with a wide flat osteotome under fluoroscopic guidance. With a medial hinge intact and easy movement of the osteotomy site, we proceed with validator placement.

### Placement of Anterior and Posterior Wedge Validators

**FIGURE 6:**
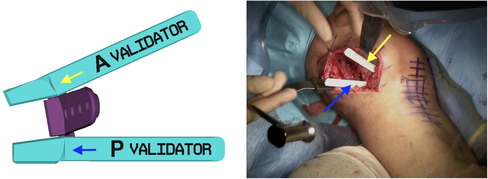
Placement of anterior and posterior osteotomy wedge validators with 3D animation. Right knee, supine position, anterior toward top right corner, posterior toward the bottom left corner. Anterior (yellow arrow) and posterior (blue arrow) wedge validators are placed by hand and malleted into place to maintain the opening wedge of the lateral distal femur osteotomy. Space must be left between the validators to allow for plate placement. (3D, 3‐dimensional.)

With the osteotome left in place, anterior and posterior wedge validators are placed by hand and malleted into place to maintain the opening wedge of the osteotomy (Figure 6). Space must be left between the validators to allow for plate placement.

### Placement of Patient‐Specific Lateral Distal Femoral Plate

**FIGURE 7:**
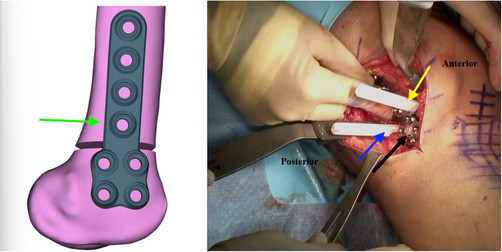
Initial placement of patient‐specific plate with 3D rendering. Right knee, supine position, anterior toward top right corner, posterior toward the bottom left corner. Fixation plate with wedge gap (black arrow) is placed into the lateral distal femur osteotomy site of the right knee between the anterior (yellow arrow) and posterior (blue arrow) wedge validators. Position of the plate on the patient can be compared with the BodyCad (Quebec City, CA) preoperative planning software position (green arrow). (3D, 3‐dimensional.)

The BodyCad (Quebec City, CA) software‐generated patient‐specific lateral distal femoral osteotomy plate with the wedge built into the plate is placed into the osteotomy site (Figure 7). A tamp and mallet are used to ensure the plate is flush with the bone. Initial plate position prior to drilling is confirmed with fluoroscopy.

### Drill Screw Holes Using Patient‐Specific Guide

**FIGURE 8:**
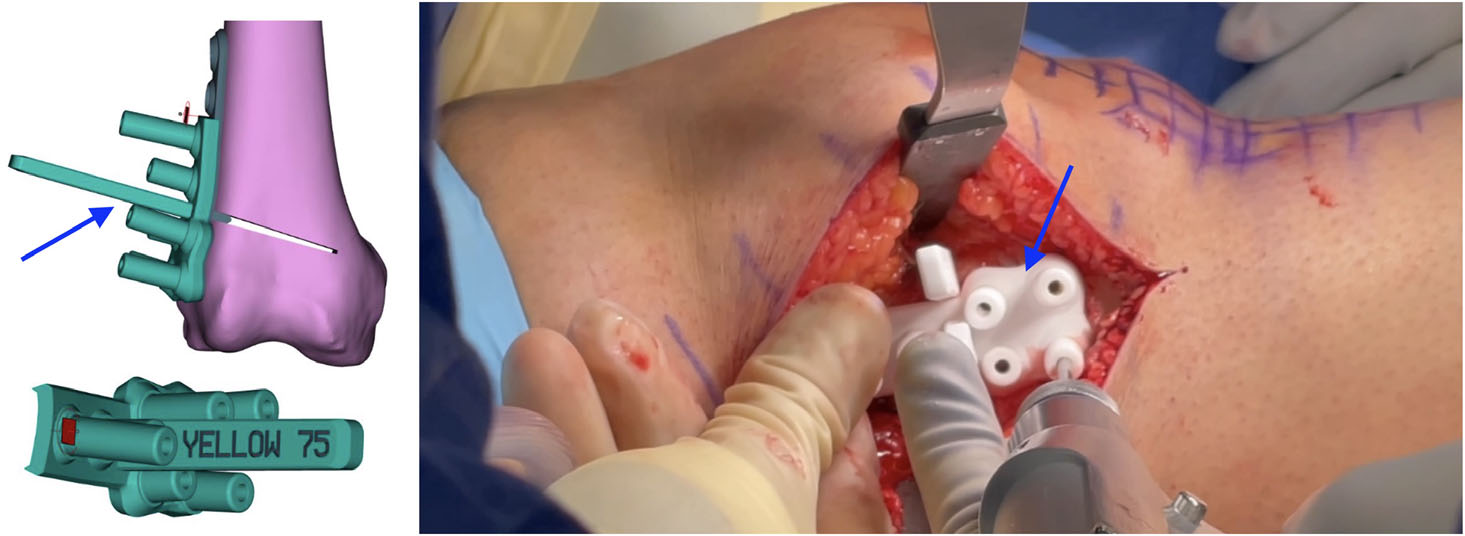
Placement of 3D drill guide with 3D rendering. Right knee, supine position, proximal to the left, distal to the right. The BodyCad (Quebec City, CA) software 3D‐printed drill guide (blue arrows) is placed over the patient‐specific lateral distal femur osteotomy plate to efficiently and accurately drill the 4 distal locking screws and two proximal screw holes nearest to the osteotomy site. (3D, 3‐dimensional.)

A custom BodyCad (Quebec City, CA) software‐generated 3D predrill guide is placed over the plate (Figure 8). Appropriate color‐coded drill bits are used to drill unicortically for the distal set of screws. A stopper on the guide prevents the drill bit from drilling bicortically. After, the 4 distal locking screws are drilled. The nearest 2 screw holes proximal to the osteotomy site are drilled bicortically. Appropriate predetermined length locking screws are used to fill the 4 distal holes first. Next, a cortical nonlocking screw is used in the proximal hole nearest to the osteotomy site to provide compression.

### Remove Validators and Fill Proximal Screw Holes

The validators are removed. The remaining proximal screw holes are filled with locking screws. A separate BodyCad (Quebec City, CA) software‐generated locking tower can be used to drill the most proximal two holes in the plate. Two bicortical locking screws are placed.

### Final Plate Fixation

**FIGURE 9:**
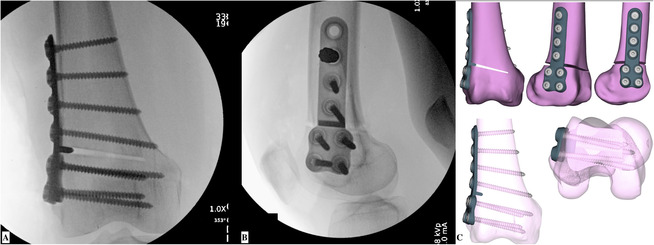
(A‐C) Final radiographic plate position with 3D rendering. Right knee, anteroposterior (A) and lateral (B) fluoroscopic images taken with patient in supine position. The position of the plate following fixation on anteroposterior (A) and lateral (B) fluoroscopic images can be compared with the preoperative projections as determined by the BodyCad (Quebec City, CA) software's 3D renderings (C). (3D, 3‐dimensional.)

Final position of the plate and screws is confirmed with fluoroscopy and compared with the 3D bone model plan (Figure 9). The tourniquet is deflated and a sterile bedside arterial Doppler assessment is performed. A medium Hemovac drain is placed and IT band closed. The incision is closed in a layered fashion. An incisional vacuum dressing is placed. The operative extremity is placed in a hinged knee brace locked in extension.

Measurements following the osteotomy are shown with specific attention paid to normalization of the MAD and lateral distal femoral angle.

### Rehabilitation

Postoperatively, the operative extremity is placed in a hinged knee brace locked in extension. Thromboprophylaxis is initiated postoperative day 1 with 81 mg of aspirin twice daily for 6 weeks. A multimodal pain regimen is used for pain control. The patient is made nonweight‐bearing of the operative extremity for 2 weeks postoperatively, with advancement to early partial weight‐bearing after radiographs at 2 weeks. The weight‐bearing status is advanced to weight‐bearing as tolerated by 6 to 8 weeks contingent on radiographic evidence of healing. For large valgus corrections, protected weight‐bearing is encouraged for up to 6 to 8 weeks postoperatively. Passive range of motion exercises begin within the first week of surgery, progressing to active motion as tolerated. Valgus stress and high‐impact activities are avoided until bony union is confirmed. Radiographic follow‐up includes X‐rays at 2, 6, and 12 weeks, and bony union is confirmed at 3 months prior to advancing to unrestricted activities.

## DISCUSSION

Recurrent lateral patellar instability following MPFL‐R occurs in up to 11% of patients and may be secondary to anatomic bony abnormalities.^3^ Genu valgum is observed in up to 60% of patients presenting with patellofemoral instability and results in an increased lateral force vector acting on the patella.^10^ Preoperative genu valgum is a known risk factor for recurrent instability and graft failure following MPFL‐R due to increased stress placed on the graft. LOW‐DFO is a surgical option that can be used in conjunction with other soft‐tissue and bony procedures to address preoperative coronal malalignment in patients with recurrent lateral patellar instability. Our technique uses PSI as an alternative to freehand osteotomy techniques to enhance the accuracy of LOW‐DFO alignment correction while attempting to mitigate the risk of intraoperative complications.

Several DFO techniques have been described including both lateral‐opening and medial‐closing wedge (MCW) osteotomies as well as those in conjunction with MPFL‐R and TTO. Case series on lateral patellar instability have shown successful clinical and radiographic outcomes in patients undergoing isolated LOW‐DFO without additional soft‐tissue procedures.^11^, ^12^ Swarup et al. studied 10 knees that underwent LOW‐DFO and showed significant improvements in patella congruency angle, MAD, as well as Visual Analog Scale and Kujala knee scores following surgery.^12^ However, correcting the underlying bony malalignment, without addressing a likely compromised MPFL, may lead to suboptimal functional outcomes with increased risk of recurrent instability. Therefore, the authors of the present technique recommend supplementing corrective DFO with MPFL reconstruction and TTO if indicated. Moran et al. performed 20 LOW‐DFO with MPFL reconstruction and found significant improvements in both radiographic and functional outcomes with 95% of knees experiencing no recurrent patellar instability.^13^ One‐half of the patients in the aforementioned study had undergone prior procedures to address patellar instability indicating the efficacy of this technique in both the primary and revision settings.

PSI has emerged as a technique to address coronal bony malalignment with enhanced precision and ease compared with traditional freehand techniques. Our technique uses preoperative computed tomography imaging and 3D reconstructions based on BodyCad (Quebec City, CA) software to create a patient‐specific cutting guide and lateral distal femoral plate in order to perform more accurate osteotomies and maximize correction precision. Much of the existing literature has highlighted the increased accuracy of patient‐specific cutting guides for DFO in the correction of coronal malalignment as well as advantages of reduced operative time and fluoroscopy use compared with freehand techniques.^6^, ^7^, ^8^, ^9^, ^14^ Arnal‐Burró et al. performed LOW‐DFO in 12 patients using PSI which was compared with a control group of 20 patients and found more accurate correction of the axial alignment, reduced operative time (32 min), and reduced used of intraoperative fluoroscopy (59 images less) in the PSI group.^6^ Jacquet et al. conducted a similar matched comparative study with 42 patients and found that the PSI group had significantly greater improvements in the accuracy of hip‐knee‐ankle angle correction measurements (0.43 ± 0.50 vs 3.95 ± 1.64, *P* < .001).^7^

Although the existing literature for PSI use with distal femoral osteotomy is limited, comparative studies using PSI in high tibial osteotomies may show additional advantages in short‐term functional outcomes compared with freehand techniques.^15^, ^16^ Gao et al. conducted a randomized control trial of 39 knees undergoing high tibial osteotomies with and without PSI for medial compartment osteoarthritis and found significantly better American Knee Society clinical scores at 3 and 6 months postoperatively in the PSI group, with no difference at final follow‐up. Similarly, Mao et al. performed a prospective comparative study and found significantly higher functional outcome scores in the PSI group at 3 months, but no significant difference at the remaining time points.^16^ Additionally, few studies have shown lower rates of intraoperative hinge fractures when using PSI compared with freehand techniques.^8^, ^9^, ^15^ Savage‐Elliott et al. conducted a comparative study of 42 patients undergoing high tibial osteotomies or DFO and noted the PSI group observed a lower rate of hinge fractures (9.5% vs 33.3%), but the differences were not found to be significant (*P* = .13).^9^ However, accurate assessment of complication rates is limited given the relatively small sample sizes present in existing studies.

Our technical note shows numerous tips (Table 1) for successful LOW‐DFO but is not devoid of limitations and disadvantages (Table 2). Disadvantages of PSI include increased implant and preoperative costs, increased lead time to surgery awaiting the manufacturing of the guide, less intraoperative flexibility, and a learning curve for surgeons unfamiliar with the technology.^8^ Additionally, our technique uses a LOW‐DFO as compared with MCW‐DFO which offers greater ability to fine‐tune intraoperative correction and may be less technically challenging.^17^ However, MCW‐DFO with MPFL‐R has been shown to provide similar efficacy and outcomes in the management of patellar instability.^18^, ^19^, ^20^ MCW‐DFO may offer advantages of more reliable bone healing and inherent stability given direct bony apposition and less risk of hardware irritation compared with LOW‐DFO techniques.^17^

**TABLE 1:** Pearls and Pitfalls of Lateral Opening Wedge Distal Femoral Osteotomy Using Patient‐Specific Instrumentation

Pearls	Pitfalls
Fluoroscopy should enter from the contralateral side, and the knee should be at resting 35° of knee flexion	Improper patient positioning and c‐arm entry from the ipsilateral side can increase the difficulty of the case
Dissection of Kaplan fibers off the distal femur allows guide placement	The patient‐specific cutting guide will not fit properly if proximal and distal Kaplan fibers are not elevated off the distal femur
Confirm the position of the hinge pins on AP and lateral fluoroscopic images and compare to the 3D computer‐generated file prior to osteotomy	Inaccuracies in cutting guide placement can lead to improper osteotomy correction
Cut the hinge pins shorter to facilitate saw entry	The oscillating saw will be blocked by the length of the pins if not shortened
Place a radiolucent Hohmann retractor posteriorly in line with the saw cutting slot in the guide	Failure to place the posterior Hohmann retractor in line with the cutting slot risks neurovascular injury of the posterior neurovascular structures
Place the drill guide over the plate to efficiently drill the most distal screw holes in the plate	If the drill guide is not used, an individual locking tower is used for each screw hole, which may increase operative and tourniquet times

3D, 3‐dimensional; AP, anteroposterior.

**TABLE 2:** Advantages and Disadvantages of Lateral Opening Wedge Distal Femoral Osteotomy Using Patient‐Specific Instrumentation

Advantages	Disadvantages
Patient‐specific plate and cutting guide allows for more accurate osteotomy gap correction	Higher implant costs, longer lead time to surgery for manufacturing of the patient‐specific implants, and additional learning curve to use the technology
Preoperative CT scanogram of the lower extremities can assess rotational profile to generate 3D bone model for more accurate osteotomy gap correction	Additional radiation burden from CT scanogram
Lateral opening distal femoral osteotomy is a reliable and reproducible method to correct genu valgum	Medial‐closing wedge distal femoral osteotomy has a more reliable rate of osteotomy union given direct bony apposition creating by the closing wedge

3D, 3‐dimensional; CT, computed tomography.

## DISCLOSURES

The author declares the following financial interests/personal relationships which may be considered as potential competing interests: G.F.R.H. reports a relationship with Arthrex that includes consulting or advisory; reports a relationship with BodyCad that includes consulting or advisory; reports a relationship with LifeNet that includes consulting or advisory. The other authors (A.P.D., E.K., N.P., A.A.) declare that they have no known competing financial interests or personal relationships that could have appeared to influence the work reported in this article.

## References

[1] Klasan A , Compagnoni R , Grassi A , Menetrey J. Promising results following derotational femoral osteotomy in patellofemoral instability with increased femoral anteversion: A systematic review on current indications, outcomes and complication rate. J Exp Orthop. 2024;11:e12032. 38774579 10.1002/jeo2.12032 PMC11106799

[2] Dixit S , Deu RS . Nonoperative treatment of patellar instability. Sports Med Arthrosc. 2017;25:72‐77. 28459749 10.1097/JSA.0000000000000149

[3] Jackson GR , Tuthill T , Gopinatth V , et al. Complication rates after medial patellofemoral ligament reconstruction range from 0% to 32% with 0% to 11% recurrent instability: A systematic review. Arthroscopy. 2023;39:1345‐1356. 36764559 10.1016/j.arthro.2023.01.098

[4] Zhang Z , Cao Y , Song G , Li Y , Zheng T , Zhang H . Derotational femoral osteotomy for treating recurrent patellar dislocation in the presence of increased femoral anteversion: A systematic review. Orthop J Sport Med. 2021;9:23259671211057130. 10.1177/23259671211057126 PMC8647269 34881342

[5] Zhang Z , Wang D , Liu Y , Wang X , Zhang H . Short‐term outcomes after derotational femoral osteotomy to correct abnormal tibiofemoral rotation in patients with recurrent patellar dislocations and severe rotational malalignment. Am J Sports Med. 2025;53:2609‐2617. 40815850 10.1177/03635465251360785

[6] Arnal‐Burró J , Pérez‐Mañanes R , Gallo‐Del‐Valle E , Igualada‐Blazquez C , Cuervas‐Mons M , Vaquero‐Martín J . Three dimensional‐printed patient‐specific cutting guides for femoral varization osteotomy: Do it yourself. Knee. 2017;24:1359‐1368. 28978460 10.1016/j.knee.2017.04.016

[7] Jacquet C , Chan‐Yu‐Kin J , Sharma A , Argenson J‐N , Parratte S , Ollivier M . More accurate correction using “patient‐specific” cutting guides in opening wedge distal femur varization osteotomies. Int Orthop. 2019;43:2285‐2291. 30413851 10.1007/s00264-018-4207-1

[8] Khela HS , Khela MS , Sriram V , Schroeder GG , Hollyer I , Sherman SL . Indications, technique, and outcomes of patient specific instrumentation for osteotomy about the knee. Curr Rev Musculoskelet Med. 2025;18:547‐557. 40632446 10.1007/s12178-025-09987-2 PMC12361020

[9] Savage‐Elliott I , Li ZI , Rao N , et al. Patient‐specific cutting guides for alignment‐correcting osteotomy about the knee: A study of accuracy, cost, and surgical and fluoroscopic safety. Orthop J Sport Med. 2025;13:23259671251339496. 10.1177/23259671251339497 PMC12280538 40697812

[10] Kirby JC , Jones H , Johnson BL , Brenner ME , Wilson PL , Ellis HB . Genu valgum in pediatric patients presenting with patellofemoral instability. J Pediatr Orthop. 2024;44:168‐173. 38014718 10.1097/BPO.0000000000002576 PMC10836788

[11] Wilson PL , Black SR , Ellis HB , Podeszwa DA . Distal femoral valgus and recurrent traumatic patellar instability: Is an isolated varus producing distal femoral osteotomy a treatment option? J Pediatr Orthop. 2018;38:e162‐e167. 29324527 10.1097/BPO.0000000000001128

[12] Swarup I , Elattar O , Rozbruch SR . Patellar instability treated with distal femoral osteotomy. Knee. 2017;24:608‐614. 28318932 10.1016/j.knee.2017.02.004

[13] Moran TE , Manley BJ , Reahl GB , et al. Lateral opening distal femoral osteotomy with concomitant medial patellofemoral ligament reconstruction is efficacious in addressing patellar instability. Arthroscopy. 2025;41:2927‐2937. 39710155 10.1016/j.arthro.2024.12.016

[14] Shi J , Lv W , Wang Y , et al. Three dimensional patient‐specific printed cutting guides for closing‐wedge distal femoral osteotomy. Int Orthop. 2019;43:619‐624. 29951692 10.1007/s00264-018-4043-3

[15] Gao F , Yang X , Wang C , et al. Comparison of clinical and radiological outcomes between calibratable patient‐specific instrumentation and conventional operation for medial open‐wedge high tibial osteotomy: A randomized controlled trial. Biomed Res Int. 2022;2022:1378042. 36467884 10.1155/2022/1378042 PMC9711981

[16] Mao Y , Xiong Y , Li Q , et al. 3D‐Printed patient‐specific instrumentation technique vs. conventional technique in medial open wedge high tibial osteotomy: A prospective comparative study. Biomed Res Int. 2020;2020:1923172. 33282939 10.1155/2020/1923172 PMC7685795

[17] Wylie JD , Maak TG . Medial closing‐wedge distal femoral osteotomy for genu valgum with lateral compartment disease. Arthrosc Tech. 2016;5:e1357‐e1366. 28149734 10.1016/j.eats.2016.08.009 PMC5263866

[18] Frings J , Krause M , Akoto R , Wohlmuth P , Frosch K‐H . Combined distal femoral osteotomy (DFO) in genu valgum leads to reliable patellar stabilization and an improvement in knee function. Knee Surg Sports Traumatol Arthrosc. 2018;26:3572‐3581. 29869201 10.1007/s00167-018-5000-9

[19] Jing L , Wang X , Qu X , et al. Closing‐wedge distal femoral osteotomy combined with medial patellofemoral ligament reconstruction for recurrent patellar dislocation with genu valgum. BMC Musculoskelet Disord. 2021;22:668. 34372805 10.1186/s12891-021-04554-5 PMC8351451

[20] Fluegel J , Zimmermann F , Gebhardt S , Milinkovic DD , Balcarek P . Combined distal femoral osteotomy and tibial tuberosity distalization is effective in patients presenting with patellar instability and patellofemoral pain due to patella alta and femoral malalignment. Arch Orthop Trauma Surg. 2023;143:2557‐2563. 35861870 10.1007/s00402-022-04541-y

